# SNORA70E promotes the occurrence and development of ovarian cancer through pseudouridylation modification of *RAP1B* and alternative splicing of *PARPBP*

**DOI:** 10.1111/jcmm.17540

**Published:** 2022-09-03

**Authors:** Shuo Chen, Qian‐hui Li, Xi Chen, Hai‐Juan Bao, Wu Wu, Fan Shen, Bing‐Feng Lu, Ru‐Qi Jiang, Zhi‐hong Zong, Yang Zhao

**Affiliations:** ^1^ Department of Obstetrics and Gynecology, Department of Gynecologic Oncology Research Office, Guangdong Provincial Key Laboratory of Major Obstetric Diseases The Third Affiliated Hospital of Guangzhou Medical University Guangzhou China

**Keywords:** alternative splicing, ovarian cancer, pseudouracil modification, *RAP1B*, SNORA70E

## Abstract

The present study demonstrated for the first time that SNORA70E, which belongs to box H/ACA small nucleolar noncoding RNAs (snoRNAs) who could bind and induce pseudouridylation of RNAs, was significantly elevated in ovarian cancer tissues and was an unfavourable prognostic factor of ovarian cancer. The over‐expression of SNORA70E showed increased cell proliferation, invasion and migration in vitro and induced tumour growth in vivo. Further research found that SNORA70E regulates RAS‐Related Protein 1B (*RAP1B*) mRNA through pseudouracil modification by combing with the pyrimidine synthase Dyskerin Pseudouridine Synthase 1 (DKC1) and increase *RAP1B* protein level. What's more, the silencing of *DKC1*/*RAP1B* in SNORA70E overexpression cells both inhibited cell proliferation, migration and invasion through reducing β‐catenin, PI3K, AKT1, mTOR, and MMP9 protein levels. Besides, RNA‐Seq results revealed that SNORA70E regulates the alternative splicing of PARP‐1 binding protein (*PARPBP*), leading to the 4th exon‐skipping in *PARPBP‐88*, forming a new transcript *PARPBP‐15*, which promoted cell invasion, migration and proliferation. Finally, ASO‐mediated silencing of SNORA70E could inhibit ovarian cancer cell proliferation, invasion, migration ability in vitro and inhibit tumorigenicity in vivo. In conclusion, SNORA70E promotes the occurrence and development of ovarian cancer through pseudouridylation modification of *RAP1B* and alternative splicing of *PARPBP*. Our results demonstrated that SNORA70E may be a new diagnostic and therapeutic target for ovarian cancer.

## 1 INTRODUCTION

Ovarian cancer is one of the three major malignant tumours of the female reproductive system. Its deep location and lack of specific screening strategies for early detection make the occurrence of ovarian cancer insidious, and metastasis may occur rapidly.^1^ Most patients present with distant metastasis at diagnosis, and their prognosis is poor, with a five‐year survival rate of approximately 29.2%, which seriously threatens the life and health of women.^2^, ^3^ Therefore, ovarian caner's molecular mechanism, occurrence, and development should be analysed, and suitable markers for early diagnosis and therapy should be identified.

Recent research has found that although noncoding RNAs (ncRNAs) lack the ability to encode proteins, they can regulate the expression or modification of proteins from multiple angles and participate in various biological functions, such as cell proliferation and migration, thus becoming a hot spot of cancer research. NcRNAs mainly include microRNAs (miRNAs), piRNAs, long noncoding RNAs (lncRNAs), circular RNAs (circRNAs), and small nucleolar RNAs (snoRNAs).^4^, ^5^

SnoRNAs are non‐coding RNAs of about 60–300 nt in size, most of which are located in nucleoli, hence their name. According to their molecular structure characteristics, snoRNAs can follow the principle of base complementary pairing to bind to their targets and induce pseudouridylation or 2'‐O‐ribose methylation to modify rRNA, tRNA, and other RNAs.^6^, ^7^ Recent studies have shown that snoRNAs are abnormally expressed in a variety of tumours and play an important role in cancer tumorigenesis and development.^8^, ^9^, ^10^ For example, SNORA23 is highly expressed in pancreatic cancer tissues. Overexpression of SNORA23 in pancreatic ductal adenocarcinoma (PDAC) cells can mediate sequence‐specific pseudouridine acidification of ribosomal RNA and by increasing the expression level of *SYNE2* (encoding spectrin repeat containing nuclear envelope protein 2), promotes pancreas carcinogenesis and development, which provides new avenues for the study of molecular mechanisms of tumorigenesis and development.^9^ However, although numerous studies have shown that ncRNAs have important functions in ovarian cancer development, we know little about snoRNAs' function and their specific regulatory mechanisms in ovarian cancer.

Our team screened snoRNAs that might function in ovarian cancer development through The Cancer Genome Atlas (TCGA) database and found that SNORA70E is an unfavourable prognostic factor for ovarian cancer. There is no gene expression data of normal ovarian tissue in the TCGA database; therefore, we further used quantitative real‐time reverse transcription PCR (qRT‐PCR) to detect the expression of SNORA70E in normal ovarian tissues and ovarian cancer tissues, and found that its expression in ovarian cancer was significantly increased. Hence, we suggested that SNORA70E might promote ovarian cancer occurrence and progression; however, the potential role and molecular mechanism of SNORA70E in tumours has not been reported.

SNORA70E belongs to the group of box H/ACA snoRNAs, which have two hairpin structures, the hinge region is box H (ANANNA), and the last 4–6 nucleotides at the 3′ end of the molecule are the highly conserved box ACA. In the human body, snoRNAs, dyskerin pseudouridine synthase 1 (DKC1), and the other three core proteins NHP2 ribonucleoprotein (NHP2), GAR1 ribonucleoprotein (GAR1), nucleolar protein 10 (NOP10) form the snoRNP complex, bind to target RNA (e.g., rRNA and tRNA) through base pairing, and participate in various pathophysiological processes through DKC1's function of catalysing the pseudouridylation of specific sites of the target RNA.^6^, ^7^ Recently, Karijolich et al.^11^ showed that mRNA can also be modified by pseudouracil. They further identified a number of pseudouracil synthetases that can act on mRNA, including DKC1. This suggested that box H/ACA snoRNAs might be combined with the principle of base complementation under the action of pseudouracil synthase DKC1 and to modify mRNA by pseudouridylation, thus changing the expression of target mRNAs or proteins, which might affect the development of tumours. In addition, studies have shown that some snoRNAs can also be processed into miRNA precursors, and form snoRNA‐like microRNAs (sno‐miRNAs) with a size of about 18–30 bp through the co‐function of DICER1, and argonaute (AGO), the key enzyme for miRNA processing and maturation. These sno‐miRNAs function as miRNAs, which mighty also lead to the silencing of downstream target genes.^12^, ^13^, ^14^

Therefore, the present study aimed to use in vitro cell experiments to detect changes in the cell cycle, apoptosis, proliferation, migration or invasion ability, and in vivo nude mouse experiments to analyse SNORA70E's molecular mechanism in ovarian cancer.

## 2 MATERIALS AND METHODS

### 2.1 Specimens of ovarian cancer

Patients who underwent gynaecological surgery at the Third Affiliated Hospital of Guangzhou Medical University (Guangzhou, China) provided specimens of ovarian cancer tissues (*n* = 70), borderline tumours (*n* = 12), benign ovarian tumours (*n* = 7), and normal ovarian tissues *(n* = 14) with patient consents. Samples were immediately frozen in liquid nitrogen. Two pathologists confirmed the tissue specimens independently. The Ethics Committee of the Guangzhou Medical University approved the study (No. 2020‐053).

### 2.2 Culture of cells and their transfection

Jennio Biotech (Guangzhou, China) or ATCC (Manassas, VA, USA) provided the human ovarian cancer cell lines (SKOV3, OVCAR3, CAOV3, and A2780). Dulbecco's modified Eagle's medium (DMEM; HyClone, Logan, UT, USA) was used to grow A2780 cells. McCoys' 5A medium was used to grow SKOV3 cells. Roswell Park Memorial Institute (RPMI)‐1640 medium (HyClone) was used to grow OVCAR3, and CAOV3 cells. Penicillin/streptomycin (100 U/ml) and 10% fetal bovine serum (FBS) were used to supplement all media. Cells were grown at 37°C in a 5% CO_2_ atmosphere. Transfection of plasmids, ASOs (amido‐bridged nucleic acid‐flanked antisense oligonucleotides), and small interfering RNA (siRNA) used Lipofectamine 3000 following to the manufacturer's protocol (Invitrogen, Carlsbad, CA, USA). A SNORA70E overexpressing plasmid was used to upregulate SNORA70E expression (TCTCTGGCTAACTAGAGAACCCACTGCTTACTGGCTTATCGAAATTAATACGACTCACTATAGGGAGACCCAAGCTGGCTAGCGTTTAAACGGGCCCTCTAGACTGCAACCAATTAAGCCGACCTAGTTCCTTTCCTCTTTGGGGCCTGGTGTTCAATAGCTGCAAACAGCAGCTTCCTTGGTAGTGTATGCAGCCTGTTTCTTGTATGGGTTGCTCTAAAGGACCTTGGAGACAGCCTCTAGATAGTTAAACCGCTGATCAGCCTCGACTGTGCCTTCTAGTTGCCAGCCATCTGTTGTTTGCCCCTCCCCCGTGCCTTCCTTGACCCTGGAAGGTGCCACTCCCACTGTCCTTTCCTAATAAAATGAGGAAATTGCATCGCATTGTCTGAGTA), and a SNORA70E ASO (GTGTTCAATAGCTGCAAACA, Ruibo, China) was used to knockdown SNORA70E expression. The sequence of si‐DKC1 was AGCCTGGATTCGACGGATA, for si‐RAP1B (targeting *RAP1B* (encoding Ras‐related protein Rap‐1b)) was ACCTAGTGCGGCAAATTAA.

### 2.3 CCK‐8 assay

Cell viability was determined using a Cell Counting Kit‐8 (CCK‐8) assay. Cells (2000 per well) were seeded in 96‐well plates and then CCK‐8 solution (10 μl per well) (Bintech Co., Ltd.) was added at 0, 24, 48, and 72 h. The plates were incubated for 2 h at 37°C in 5% CO_2_. A microplate spectrophotometer (BioTek Instruments, Winooski, VT, USA) was then used to measure the absorbance at 450 nm.

### 2.4 Cell proliferation analysis using EdU assay

Cells were seeded in 96‐well plates, after which cell proliferation was measured using a Click‐iT™ Plus EdU Alexa Fluor™ 555 Imaging Kit (Invitrogen) under the guidance of manufacturer's protocol.

### 2.5 Assay of apoptosis

Collected cells were resuspended in 100 μl of 1 × buffer with fluorescein isothiocyanate (FITC)‐labelled annexin V and PE or 7AAD and PE (5 μl each, BD) and incubated for 15 min in the dark. Thereafter, cells were added with buffer (400 μl) and flow cytometry was carried out within 1 h to determine the rate of cell apoptosis.

### 2.6 Assessment of wound healing

A wound‐healing assay was used to assess cell migration. Cells at 6 × 10^5^ cells per well were seeded in 6‐well plates. After the cells grew to 80% confluence, a vertical wound was made in the confluent cell layer using a 200‐μl pipette tip. Three washes with phosphate‐buffered saline (PBS) were used to remove excess cells. To each well, 2 ml of serum‐free medium was added and the cells were incubated at 37°C in 5% CO_2_. Images of the wound were acquired under an optical microscope at 0, 24, and 48 h. Image J software (National Institutes of Health, Bethesda, MD, USA) was then used to calculate the size of the wound area. The following equation was used to calculate the wound‐healing rate: (original wound area—wound area at each time point)/original wound area × 100%.

### 2.7 Assay of cell invasion

Transwell chambers were used to assess cell invasion. Serum‐free medium was used to dilute Matrigel matrix (BD Biosciences, San Jose, CA, USA) 1:10, which was added to the upper Transwell chamber. The whole Transwell apparatus was placed in an incubator for 3–4 h for coagulation. Thereafter, 50,000 cells in serum‐free medium (200 μl) were added to the upper chamber, and as a chemoattractant, complete cell culture medium (600 μl) was placed the lower chamber. After incubation for 48 h, three washes of the chambers with PBS were performed, and the cells in the lower chamber were fixed for 15 min using 5% paraformaldehyde, before being stained for 15 min with crystal violet solution. Finally, cell invasion was evaluated by counting the cells under a microscope (Olympus, Tokyo, Japan).

### 2.8 Quantitative real‐time reverse transcription PCR (qRT‐PCR)

The TRIzol reagent (Takara, Shiga, Japan) was used to extract total RNA from cells and tissues. The TRIzol mixture (1 ml) was added with chloroform (200 μl), mixed, and centrifuged. The upper aqueous phase was removed into a fresh tube, and the RNA was precipitated by the addition of an equal volume of isopropanol. The sample was centrifuged, the supernatant was discarded, and the pellet was washed using 75% ethanol. After drying, diethyl pyrocarbonate (DEPC) water was used to dissolve the precipitate. An ultraviolet spectrophotometer (Unico, Shanghai, China) was used to determine the OD value at 260 nm to calculate the RNA concentration. Then, cDNA was synthesized from the RNA using reverse transcription following the supplier's protocol (Takara). The cDNA was then used as the template in a real‐time quantitative PCR reaction, performed using a SYBR PrimeX EX‐TAQ Patent II Kit (Takara). Finally, to assess the target gene's relative expression, the cycle threshold (Ct) values of the control *GAPDH* (encoding glyceraldehyde‐3‐phosphate dehydrogenase) gene, and the target gene were compared according to the 2^−ΔΔCt^ method (GenePharma, Shanghai, China).

### 2.9 Western blotting

Radioimmunoprecipitation assay (RIPA) buffer with protease inhibitors was used to lyse the cells. The total proteins were quantified, and 40 μg of denatured protein was subjected to 10% sodium dodecyl sulfate‐polyacrylamide gel electrophoresis (SDS‐PAGE), and then the separated proteins were electro‐transferred onto a methanol pre‐activated polyvinylidene difluoride (PVDF) membrane. After washing the membrane for 2 min with Tris‐buffered saline and Tween 20 (TBST), 3% bovine serum albumin (BSA) was used to block non‐specific binding by incubation for 2 h at room temperature. Thereafter, primary antibodies against *RAP1B*, β‐catenin, phosphatidylinositol‐4,5‐bisphosphate 3‐kinase (PI3K), matrix metalloproteinase 9 (MMP9), protein kinase B (AKT), mammalian target of rapamycin (mTOR) (1:1000, Proteintech Group, Chicago, IL, USA), and GAPDH, α‐Tublin, β‐actin (1:10000, Proteintech Group) were incubated with the membrane at 4°C overnight. Next day, TBST was used to wash the membrane three times and then anti‐rabbit/mouse secondary antibodies were incubated with the membrane for 1.5 h at room temperature. After another three washes with TBST, the ECL system (Santa Cruz Biotechnology, Santa Cruz, CA, USA) was used to visualize the immunoreactive proteins on the membrane.

### 2.10 RNA binding protein immunoprecipitation (RIP) assay

The RIP assay used a Magna RIP RNA‐Binding Protein Immunoprecipitation Kit (Millipore, Bedford, MA, USA). Briefly, RIP lysis buffer was used to lyse cells, and the resultant cell extract was incubated with RIP buffer comprising magnetic beads conjugated with human anti‐AGO2 antibodies, anti‐DKC1 antibodies, anti‐RAP1B antibodies, or normal rabbit IgG (negative control). Thereafter, the samples were incubated with proteinase K to digest the proteins. Then, we isolated the immunoprecipitated RNA and subjected it to qRT‐PCR analysis.

### 2.11 Detection of pseudouridine levels

High performance liquid chromatographic instruments manufactured by Waters (Milford, MA, USA) were used for detection. The chromatographic column was ECOSIL C185 μm 4.6 mm × 250 mm, and the mobile phase was 2.5 mM ammonium acetate (PH = 4.0) buffer containing 5% acetonitrile. The detection wavelength was 263 nm, the flow rate was 0.8 ml/min, the column temperature was 25°C, and the sample injection was 10 μl. A pseudouridine reference standard was accurately weighed, and double distilled water was added to prepare a solution containing 2.7 mg per 1 ml, which was used as the reference standard stock solution. An appropriate amount of pseudouridine stock solution was accurately aspirated and successively diluted with re‐distilled water, to prepare a range of standard solutions containing pseudo‐uridine, and 10 μl was precisely aspirated, injected, and the chromatogram was recorded. The chromatographic conditions were observed, and the chromatograms and peak areas were recorded. The standard curve was plotted with peak area (A) as the vertical coordinate and injection concentration (μg/ml) as the horizontal coordinate. A 500 μl sample was centrifuged at 12,000 × *g* for 20 min at 4°C, and the supernatant was collected. The sample was measured according to the above chromatographic conditions, the chromatographic diagram and the peak area were determined, and the content of the pseudouridine in the sample was calculated using the results for the standards and a regression equation.

### 2.12 RNA‐Seq analysis

CAOV3 cells were transfected with the SNORA70E overexpression plasmid or empty vector. Total RNAs were isolated using Trizol (Takara, Japan). For the RNA sample preparations, 1 μg RNA per sample was used as input material. An Neb Next Ultra RNA Library Prep Kit for Illumina (Neb, Ipswich, MA, USA) was used to generate the sequencing libraries following the supplier's protocol. In addition, an index code was added to determine the sequence attributes of each sample. Briefly, mRNA was purified, cleaved at elevated temperature, and cDNA was synthesized. An AMPure XP system (Beckman Coulter, Beverly, CA, USA) was used to purify the library fragments, and those with a length of 150–200 bases were screened. PCR was carried out using universal primers, index primers, and polymerase. Finally, the AMPure XP system was used to purify the PCR products and an Agilent Bioanalyzer 2100 system (Agilent, Santa Clara, CA, USA) was used to evaluate the quality of the library. The cBot Cluster Generation System was used to cluster the index‐coded samples employing a TruSeq PE Cluster Kit v3‐cBot‐HS (Illumina, San Diego, CA, USA) following the supplier's protocols. Thereafter, the Illumina NovaSeq platform was used to sequence the prepared libraries, generating 150 bp paired end reads.

### 2.13 Fluorescence in situ hybridization (FISH) assay

FISH assay was performed following the manufacturer's instructions (GenePharma, Shanghai, China), as we had introduced before.^15^ The probe used for SNORA70E was 5′–GGGTAAAACTCCCTACCTGGTGTCTCCGT–3′ (Hanbio Biotechnology, China).

### 2.14 Assay of subcutaneous tumour dissemination

Vital River Laboratories (Beijing, China) supplied the BALB/c nude mice, which were housed in a specific pathogen‐free (SPF) environment. SNORA70E overexpressing CAOV3 cells or control CAOV3 cells (1 × 10^7^ in 150 μl of FBS‐free media) were injected subcutaneously into 5‐week‐old female mice to establish a subcutaneous dissemination model (*n* = 6). Mice were euthanized at various times post‐injection, the tumour nodes were resected, and their volumes were measured. Besides, OVCAR3 cells (5 × 10^6^ in 150 μl of FBS‐free media) were injected subcutaneously into 5‐week‐old female mice (*n* = 12), when most of the tumour volume reached 5 mm in diameter, the mice were randomly divided into two treatment groups, followed by injection with 250 mg/kg/week of ASO‐SNORA70E or ASO‐NC administered subcutaneously for 4 weeks. The Guangzhou Medical University Animal Care and Use Committee approved the animal experiments. The Guide for the Care and Use of Laboratory Animals (published by the National Institute of Health) was followed when carrying out the animal experiments.

### 2.15 Statistical analysis

SPSS 22.0 software (IBM Corp., Armonk, NY, USA) was used to perform the statistical analyses, the Mann–Whitney U test and paired samples *t*‐test were used to compare the means of different groups. Three repeats were performed for each experiment, and the overall parameters for each group of data were represented by the mean ± SD. Statistical significance was accepted at a *p*‐value <0.05.

## 3 RESULTS

### 3.1 The relationship between ovarian cancer clinicopathological characteristics and the expression level of SNORA70E

**FIGURE 1:**
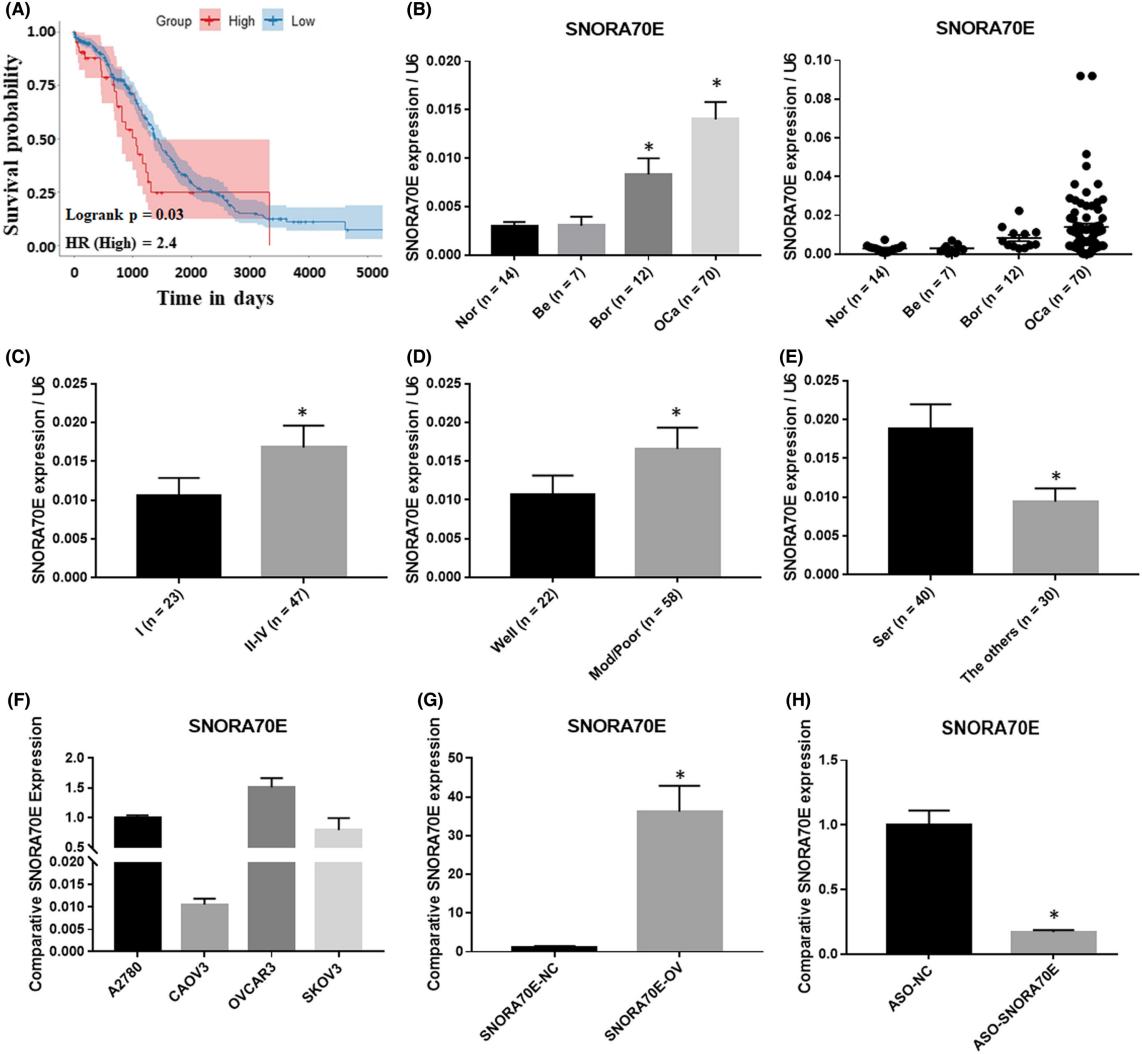
SNORA70E expression in ovarian tissues and cancer cell lines. SNORA70E is an unfavourable prognostic factor for ovarian cancer (A). SNORA70E expression was significantly higher in epithelial ovarian cancer tissues than in borderline tumour tissues, benign tissues, and normal ovaries (B). And SNORA70E expression was higher in stages II–IV than in stage I (C); in the moderately and poorly pathological classified disease than in pathologically well‐classified disease (D); in ovarian serous adenocarcinoma than in the other types (E). Data is calculated and shown as the mean ± SD (error bars). *p < 0.05. (Mann–Whitney U test). SNORA70E expression in ovarian cancer cell lines (F). SNORA70E overexpression induced SNORA70E expression level in CAOV3 cells (G), SNORA70E downregulation by ASO reduced SNORA70E expression level in OVCAR3 cells (H). Data is calculated and shown as the mean ± SD (error bars) from three independent repeats. *p < 0.05. (Student's *t*‐test).

According to the TCGA database, SNORA70E is an unfavourable prognostic factor for ovarian cancer (Figure 1A). Thus qRT‐PCR was used to assess the expression of SNORA70E in ovarian carcinoma tissues, borderline tumour tissues, benign tissues, and normal ovarian tissues. SNORA70E expression was significantly higher in epithelial ovarian cancer tissues than in borderline tumour tissues, benign tissues, and normal ovaries (Figure 1B, *p* < 0.05, Table S1). Compared with that in stage I disease, SNORA70E expression was higher in stages II–IV (Figure 1C, *p* < 0.05). In addition, compared with that in pathologically well classified disease, the SNORA70E expression was higher in the moderately and poorly pathological classified disease (Figure 1D, *p < 0.05), besides, SNORA70E expression was higher in ovarian serous adenocarcinoma than in the other types (Figure 1E, *p < 0.05, Table S2). These results indicated that SNORA70E participates in ovarian cancer tumorigenesis and progression, and might be related to poor prognosis.

### 3.2 The expression of SNORA70E in multiple ovarian cancer cell lines

In order to choose suitable cell lines for in vitro experiments, qRT‐PCR was used to assess the expression of SNORA70E in four ovarian cancer cell lines. SNORA70E expression was lowest in CAOV3 cells and highest in OVCAR3 cells (Figure 1F). Therefore, we used CAOV3 cells for transfection with SNORA70E overexpression plasmid and OVCAR3 cells for transfection with SNORA70E ASO. The transfection efficiency was confirmed using qRT‐PCR (Figure 1G,H, *p < 0.05).

### 3.3 The effects on ovarian cancer cells of SNORA70E overexpression in vivo and in vitro

**FIGURE 2:**
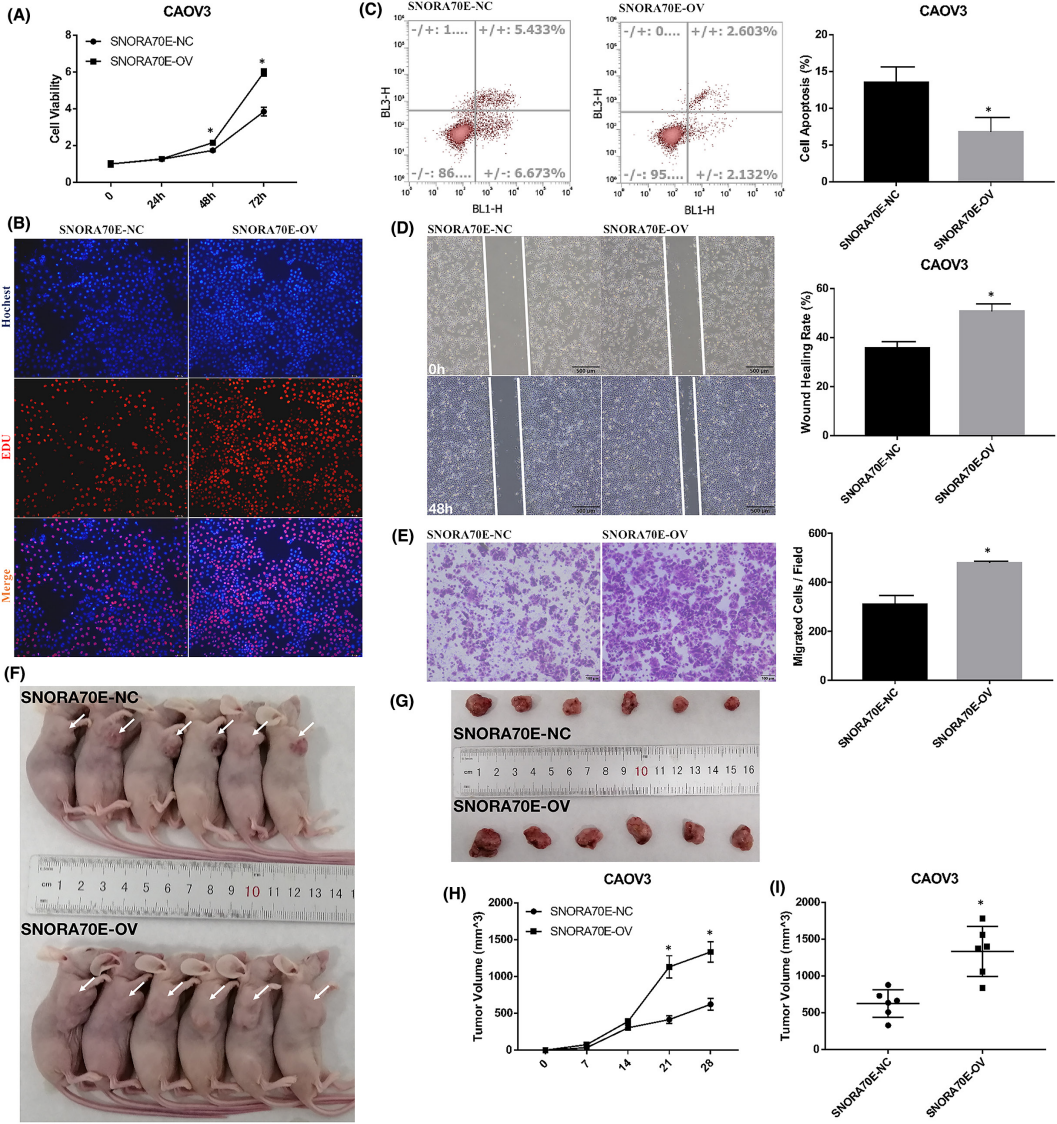
The effects on ovarian cancer cells of SNORA70E overexpression both in vivo and in vitro. SNORA70E overexpression in vitro induced cell proliferation (A–B); decreased cell apoptosis (C); and induced cell migration (D) and invasion (E). SNORA70E overexpression in vivo induced tumorigenicity (F–H), with larger tumour volumes (I). Data is calculated and shown as the mean ± SD (error bars) from three independent repeats. *p < 0.05. (Student's *t*‐test).

To assess invasion, migration, and proliferation of ovarian cancer cells, Transwell, wound healing, cell apoptosis, and CCK‐8 assays were used. The overexpression of SNORA70E in CAOV3 cells induced cell proliferation (Figure 2A,B); decreased cell apoptosis (Figure 2C, *p* < 0.05); and induced cell migration and invasion (Figure 2D,E, *p* < 0.05). When mice were injected with CAOV3 cells overexpressing SNORA70E, compared with that in the control group (Figure 2F,G), tumorigenicity was induced (Figure 2H, *p* < 0.05), with larger tumour volumes (Figure 2I, *p* < 0.05). These results indicated that SNORA70E promotes ovarian cancer tumorigenesis and progression.

### 3.4 The effect of SNORA70E downregulation in ovarian cancer cells

**FIGURE 3:**
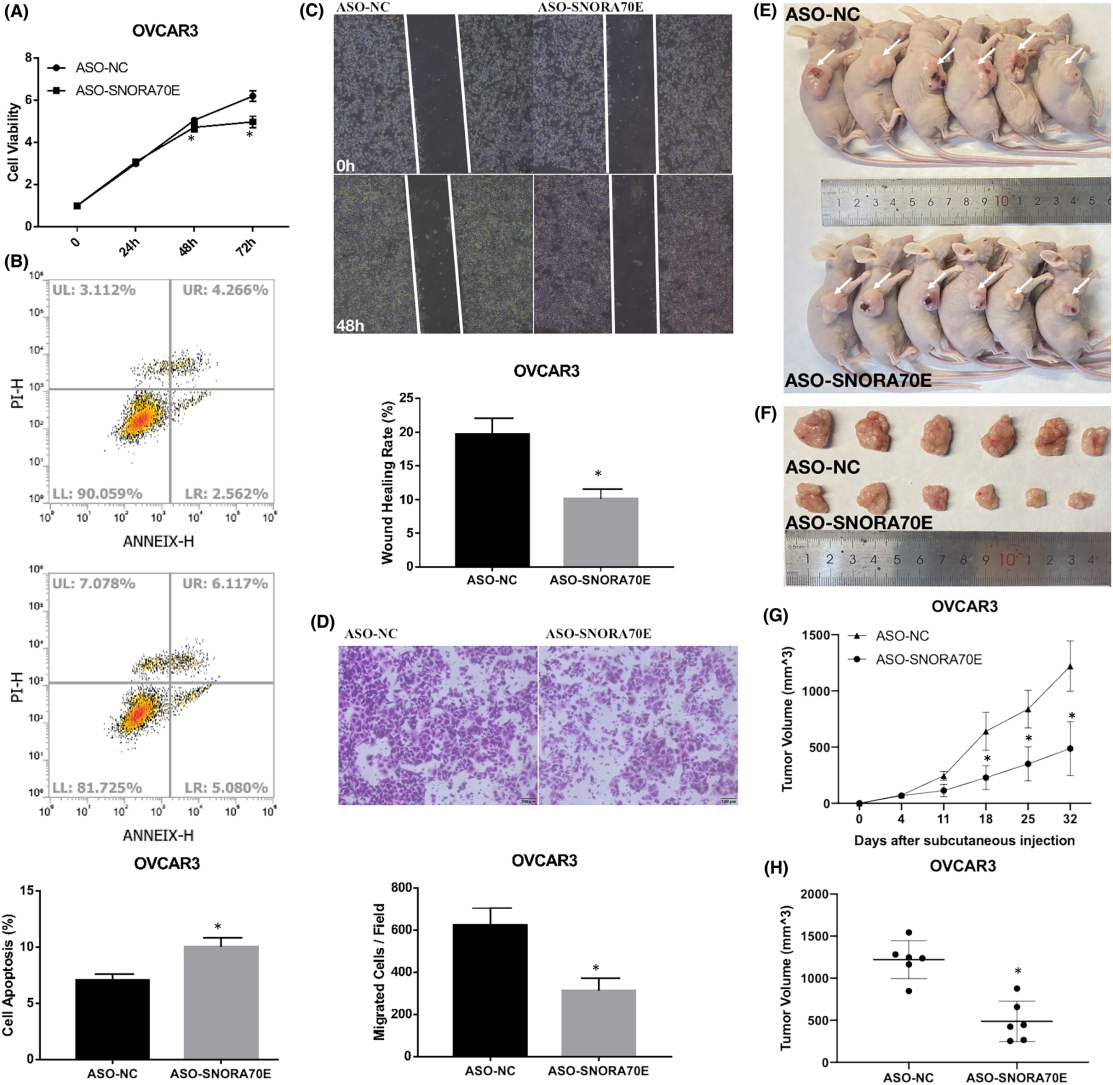
The effect of SNORA70E downregulation in ovarian cancer cells. SNORA70E downregulation in OVCAR3 cells inhibited cell proliferation (A), increased cell apoptosis (B), decreased cell migration (C) and invasion (D). ASO‐SNORD70E injection subcutaneously reduced tumorigenicity (E–G), with smaller tumour volumes (H). Data is calculated and shown as the mean ± SD (error bars) from three independent repeats. *p < 0.05. (Student's *t*‐test).

We further confirmed the function of SNORA70E through designing a specific ASO, which could downregulate SNORA70E expression. Our results showed that when SNORA70E was downregulated in OVCAR3 cells, compared with those in the negative control, cell proliferation was inhibited significantly (Figure 3A, *p* < 0.05). There was an increases in cell apoptosis (Figure 3B, *p* < 0.05), but a decrease in migration and invasion (Figure 3C,D, *p* < 0.05). In vivo results showed that compared with that in the ASO‐NC group, tumorigenicity was reduced after ASO‐SNORD70E injection subcutaneously (Figure 3E–G, *p* < 0.05), with smaller tumour volumes (Figure 3H, *p* < 0.05). Suggesting that SNORA70E may be a new diagnostic and therapeutic target in ovarian cancer.

### 3.5 The cellular location of SNORA70E and differential protein levels after SNORA70E overexpression

**FIGURE 4:**
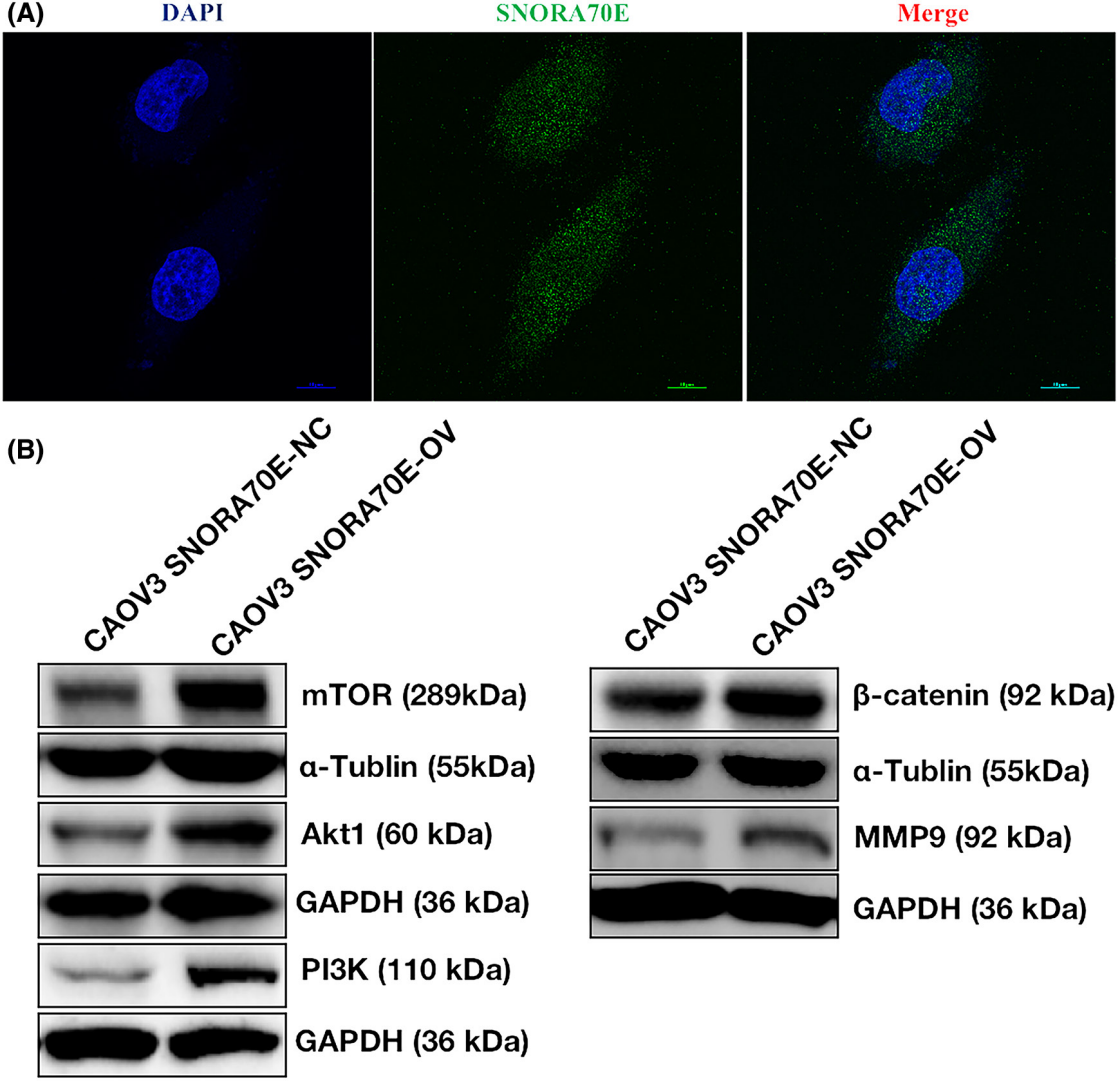
The cellular location of SNORA70E and differential protein levels after SNORA70E overexpression. SNORA70E locates both in nucleus and in cytoplasm (A). SNORA70E overexpression increased β‐catenin, PI3K, AKT1, mTOR, and MMP9 protein levels (B).

In order to konw the molecular mechanism of SNORA70E, we first checked the cellular lacation through FISH, we found that SNORA70E not only locates in nuclear but also in cytoplasm (Figure 4A). We further overexpressed SNORA70E in CAOV3 cells and performed western blotting. The results showed that SNORA70E overexpression increased β‐catenin, PI3K, AKT1, mTOR, and MMP9 protein levels (Figure 4B). These results indicated that SNORA70E could regulate β‐catenin, PI3K, AKT1, mTOR, and MMP9 protein expression.

### 3.6 SNORA70E binds to the pyrimidine synthase DKC1

**FIGURE 5:**
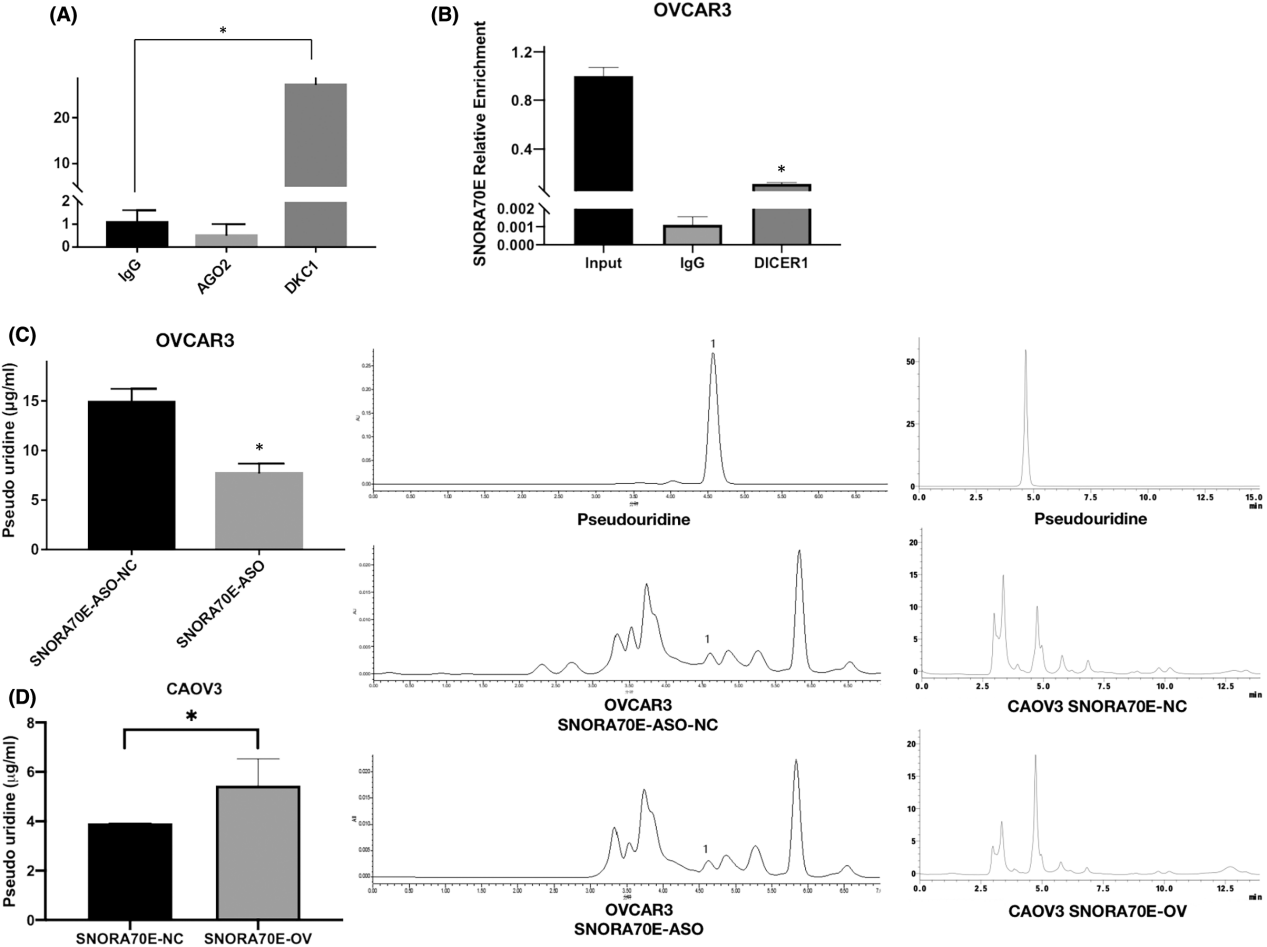
SNORA70E binds to the pyrimidine synthase DKC1. SNORA70E does not combine with AGO2, but bound to DKC1 (A) and DICER1 (B), SNORA70E downregulation decreased the level of pseudouridine‐modified metabolite pseudouridine (C), SNORA70E overexpression increased the level of pseudouridine‐modified metabolite pseudouridine (D). Data is calculated and shown as the mean ± SD (error bars) from three independent repeats. *p < 0.05. (Student's *t*‐test).

Studies had reported that box H/ACA snoRNAs might combine with pseudouracil synthase DKC1 and to modify mRNA by pseudouridylation, besides, it may also process into miRNA precursors and form sno‐miRNA through the co‐function of DICER and AGO. Through RIP experiments, we found that SNORA70E combine with DICER but not with AGO2, which means that SNORA70E might have a “miRNA like” function but need further research. Besides, we found that SNORA70E bound to DKC1, the pyrimidine synthase (Figure 5A,B, *p* < 0.05), and we further found that after silencing SNORA70E expression, the level of pseudouridine‐modified metabolite pseudouridine was decreased significantly, while overexpression of SNORA70E in CAOV3 cells could increase the level of pseudouridine‐modified metabolite pseudouridine (Figure 5C,D, *p* < 0.05). These results indicated that SNORA70E may combine with DKC1 to modify mRNA by pseudouridylation, thus influence the expression of target mRNAs or proteins.

### 3.7 Silencing DKC1 could reverse SNORA70E's oncogenic role

**FIGURE 6:**
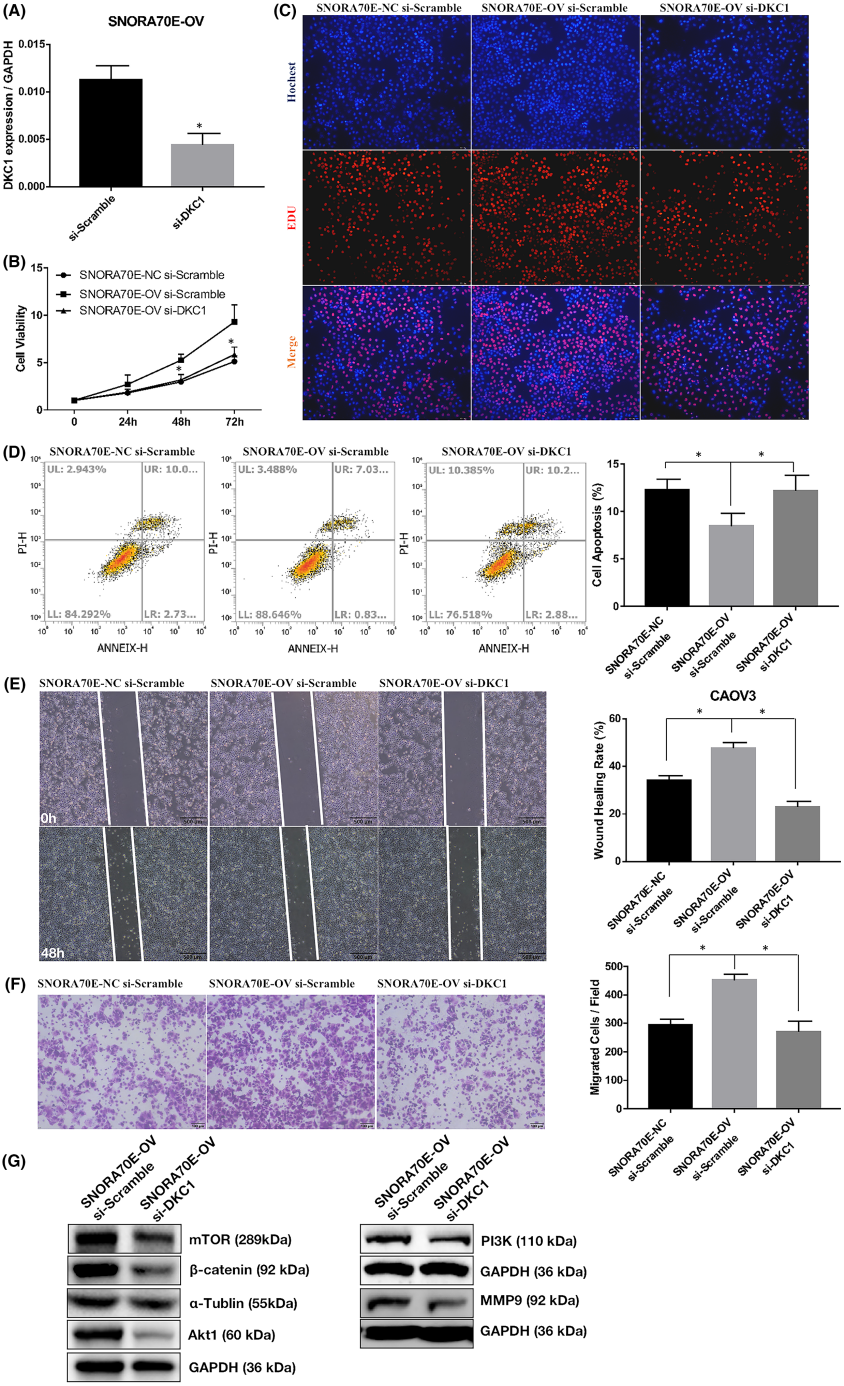
Silencing DKC1 could reverse SNORA70E's oncogenic role. Silencing *DKC1* in SNORA70E‐overexpressing CAOV3 cells (A) reduced cell proliferation (B–C), promoted apoptosis (D), inhibited cell migration (E) and invasion (F), decreased β‐catenin, PI3K, AKT1, mTOR, and MMP9 protein levels (G). Data is calculated and shown as the mean ± SD (error bars) from three independent repeats. *p < 0.05. (Student's *t*‐test).

In SNORA70E‐overexpressing CAOV3 cells, silencing *DKC1* (Figure 6A, *p* < 0.05) reduced cell viability (Figure 6B, *p* < 0.05), and cell proliferation (Figure 6C) promoted apoptosis (Figure 6D, *p* < 0.05) and inhibited cell migration and invasion (Figure 6E,F, *p* < 0.05), and siliencing *DKC1* in OVCAR3 cells whose basic SNORA70E expression is high had the same function (Figure S1). Besides, silencing *DKC1* in SNORA70E‐overexpressing CAOV3 cells also decreased β‐catenin, PI3K, AKT1, mTOR, and MMP9 protein levels (Figure 6G).

### 3.8 SNORA70E binds and regulates *RAP1B* expression

**FIGURE 7:**
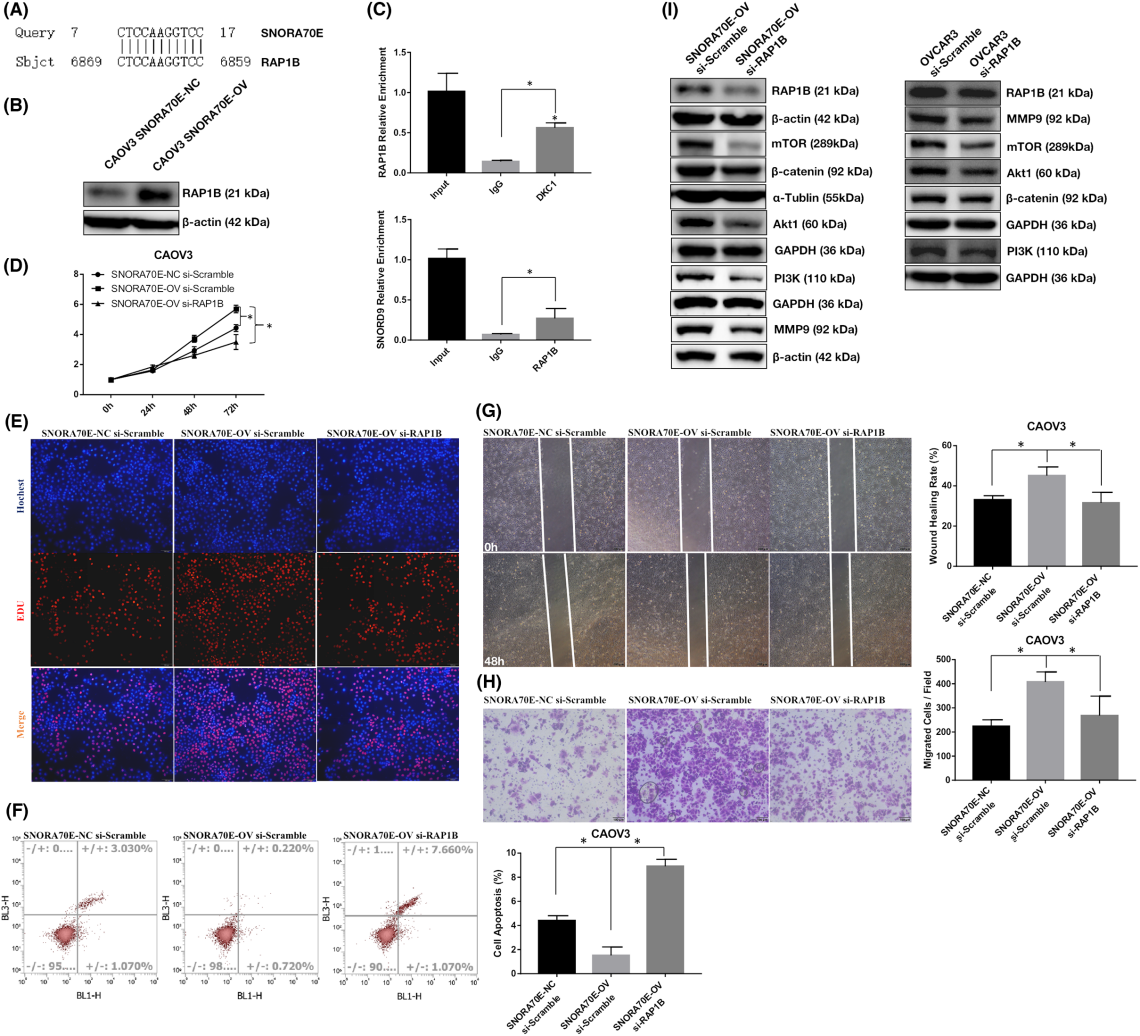
SNORA70E binds and regulates *RAP1B* expression. There is a binding site between SNORA70E and *RAP1B* (A). The overexpression of SNORA70E increased the *RAP1B* protein level (B). DKC1 bind with *RAP1B* mRNA (C). Silencing *RAP1B* in SNORA70E overexpression cells inhibited cell proliferation (D–E), migration (G), invasion (H), and induced apoptosis (F), reduced the levels of *RAP1B*, β‐catenin, PI3K, AKT1, mTOR, and MMP9 proteins (I). Data is calculated and shown as the mean ± SD (error bars) from three independent repeats. *p < 0.05. (Student's *t*‐test).

Box H/ACA snoRNAs can form complementary pairs with modified sites on the target RNA by forming a SNORNP complex with DKC1. Through BLAST sequence alignment, we found that there is a binding site between SNORA70E and *RAP1B* (Figure 7A). The overexpression of SNORA70E increased the *RAP1B* protein level significantly (Figure 7B), and RIP detection found that DKC1 can bind to *RAP1B* mRNA (Figure 7C, *p* < 0.05). We also found that both silencing *RAP1B* in SNORA70E overexpression CAOV3 cells and in OVCAR3 cells whose basic SNORA70E expression is high inhibited cell proliferation, migration, and invasion, and induced apoptosis (Figure 7D–H, Figure S1), and reduced the levels of *RAP1B*, β‐catenin, PI3K, AKT1, mTOR, and MMP9 proteins (Figure 7I). Besides, siliencing *β‐catenin, PI3K, and MMP9* in SNORA70E overexpression CAOV3 cells also inhibited cell proliferation, migration, and invasion, and induced apoptosis (Figure S2). These results indicated that SNORA70E could promote the occurrence and development of ovarian cancer through pseudouridylation modification of *RAP1B*.

### 3.9 SNORA70E regulates the alternative splicing of *PARPBP*

**FIGURE 8:**
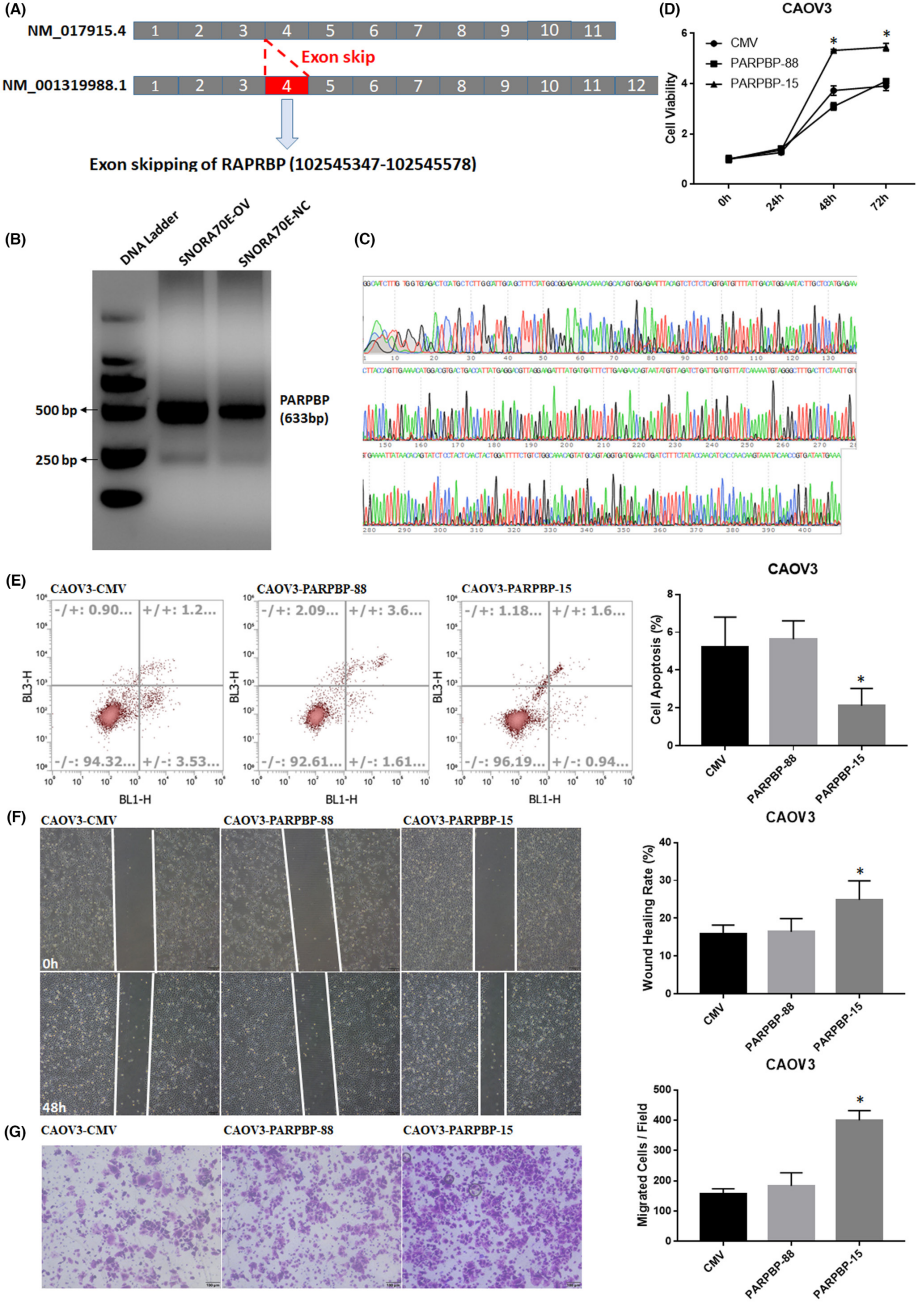
SNORA70E regulates the alternative splicing of *PARPBP*. SNORA70E regulated the alternative splicing of *PARPBP* (A). PCR results confirmed this Exon skipping event after SNORA70E overexpression (B–C). *PARPBP‐15* overexpression promoted cell proliferation (D), migration (F), and invasion (G), inhibited cell apoptosis (E). Data is calculated and shown as the mean ± SD (error bars) from three independent repeats. *p < 0.05. (Student's *t*‐test).

The RNA‐Seq results revealed that SNORA70E regulated the alternative splicing (AS) of *PARPBP* (encoding PARP1 binding protein): The 4th Exon was lost in NM_001319988.1 (*PARPBP‐88*), forming a new transcript NM_017915.4 (*PARPBP‐15*) (Figure 8A). PCR results confirmed this Exon skipping event after SNORA70E overexpression (Figure 8B–C). Further studies showed that the overexpression of *PARPBP‐15* promoted ovarian cancer cell proliferation (Figure 8D, *p* < 0.05), migration (Figure 8F, *p* < 0.05), and invasion (Figure 8G, *p* < 0.05), inhibited cell apoptosis (Figure 8E, *p* < 0.05), while the overexpression of *PARPBP‐88* did not. Besides, *PARPBP‐15* overexpression in SNORA70E‐ASO transfected OVCAR3 cell lines could significantly reversed the inhibition ability by SNORA70E downregulation (Figure S3). Above, these results suggested that SNORA70E‐regulated alternative splicing of *PARPBP* induced ovarian tumorigenesis and progression.

## 4 DISCUSSION

To identify markers for the diagnosis and treatment of epithelial ovarian cancer, we screened small noncoding RNAs in the TCGA database, and found that the H/ACA box‐type snoRNA, SNORA70E, which pseudouridylates ribosomal RNAs in a sequence‐specific manner, is an unfavourable prognostic factor for ovarian cancer. We further checked SNORA70E in our own clinical samples, and found that compared with those in normal ovaries, the SNORA70E expression levels were markedly increased in epithelial ovarian cancer tissues, and was associated with FIGO stage and differentiation, which suggested that SNORA70E participates in ovarian cancer tumorigenesis and progression, and might be related to poor prognosis.

We assessed SNORA70E's function in ovarian cancer cells using in vivo and in vitro assays. CAOV3 cells transfected with the SNORA70E overexpression plasmid showed reduced cell apoptosis and increased cell invasion, migration, and proliferation, besides, SNORA70E overexpression induced tumour growth in vivo. However, in ovarian cancer, what is the mechanism by which SNORA70E affects tumorigenesis and progression?

Recent studies showed that gene expression can be regulated by snoRNAs via gene‐related ribosome modulation or by snoRNA‐derived miRNA‐like molecules in the cytoplasm.^16^, ^17^ Target genes are regulated by an RNA‐induced silencing complex comprising argonaute 2 (Ago2) and the miRNA. However, RIP experiments showed that SNORA70E does not combine with AGO2, but does combine with DKC1, the pyrimidine synthase. Moreover, after silencing SNORA70E expression, the level of pseudouridine‐modified metabolite pseudouridine decreased significantly. Silencing *DKC1* expression in SNORA70E stably overexpressing ovarian cancer cells inhibited the increased cell proliferation and migration ability. Thus, we suggest that SNORA70E might promote ovarian cancer tumorigenesis and progression through pseudouridine modification of downstream genes.

Recent studies have shown that mRNA can also be modified by pseudouracil, and a number of pseudouracil synthetases that can act on mRNA have been identified, including DKC1.^8^ In a BLAST search for downstream genes complementary to the SNORA70E sequence, we found that there may be binding sites between SNORA70E and *RAP1B*, which functions as a tumour promoter. *RAP1B* is mainly located in the nucleus, and studies have shown that *RAP1B* expression is associated with tumorigenesis and metastasis in, for example, ovarian cancer, oesophageal squamous cell carcinoma, and gastric cancer, and can act as a tumour promoter by regulating multiple signalling pathways such as Wnt/β‐catenin, PI3K/AKT/mTOR pathway.^18^, ^19^, ^20^, ^21^, ^22^ We confirmed that the overexpression of SNORA70E induced the expression of the *RAP1B* protein significantly. Through RIP detection, we found that DKC1 can bind to *RAP1B* mRNA, thus we suggest that SNORA70E regulates *RAP1B* mRNA through pseudouracil modification, affecting its protein expression.

Silencing of *RAP1B* in SNORA70E overexpressing CAOV3 cells or high expression OVCAR3 cells inhibited cell proliferation, migration and invasion, increased apoptosis, and decreased the levels of β‐catenin, PI3K, AKT1, mTOR, and MMP9. Studies have reported that Wnt/β‐catenin signalling pathways play an active role in cancer stem cells (CSCs) and carcinogenesis in many tumours, including ovarian cancer subtypes.^23^ Besides, the cell survival, growth, and proliferation of ovarian cancer id regulated by the PI3K/AKT/mTOR signalling pathway.^24^ Thus, we suggest that SNORA70E might regulate *RAP1B* and further regulates the expression of β‐catenin, PI3K, AKT1, mTOR, and MMP9 to exert cancer‐promoting effects.

RNA variable shearing is an important component of eukaryotic gene expression regulation. That is, the exon of RNA generated by the main gene or mRNA precursor transcription is reinable by RNA shearing, thereby producing different mRNAs, which might play different effects. The RNA‐Seq results showed that in SNORA70E‐regulated alternative splicing (AS) of *PARPBP*, the 4th Exon was lost in *PARPBP‐88*, forming transcript *PARPBP‐15*, which was confirmed using PCR. Alternative splicing refers to the process by which the exons of the RNA transcribed from the main gene or mRNA precursor are variously rearranged via RNA splicing. The resulting different mRNAs may be translated into different protein constructs. Therefore, a gene might encode multiple proteins. There are seven types of AS, including mutually exclusive exons (ME), alternate terminator (AT), alternate promoter (AP), alternate acceptor site (AA), alternate donor site (AD), retained intron (RI), and exon skipping (ES). Alternative splicing occurs when different exons or introns are retained or excluded to generate alternative mRNA transcripts, and this process significantly increases the proteome diversity and cell complexity.^25^, ^26^ AS profoundly alters the function of proteins by changing their stability, adding or deleting structural domains, and modifying their protein–protein interactions.^27^ AS has been increasingly implicated in human diseases, especially cancer.^28^ The alternative splicing of genes modifies proteins involved in many malignant activities, including proliferation, invasion, metastasis, apoptosis, hypoxia, metabolic changes, and immune escape.^29^ Aberrant alternative splicing is a potential biomarker of tumorigenesis and prognosis and is also a therapeutic target in malignancy.^30^, ^31^ Further studies showed that the overexpression of *PARPBP‐15* could promote the invasion, migration, and proliferation of ovarian cancer cells, while *PARPBP‐88* could not. Besides, *PARPBP‐15* overexpression in SNORA70E‐ASO transfected OVCAR3 cell lines could significantly reversed the inhibition ability by SNORA70E‐ASO. Above, these results suggested that SNORA70E‐regulated AS of *PARPBP*‐induced ovarian tumorigenesis and progression.

Furthermore, ASO‐mediated silencing of SNORA70E could inhibit cell proliferation, invasion, migration ability and induce apoptosis in vitro, and inhibit tumorigenicity in vivo, suggesting that SNORA70E may be a new diagnostic and therapeutic target in ovarian cancer.

In conclusion, SNORA70E, which is highly expressed in ovarian cancer, regulates *RAP1B* mRNA through pseudouracil modification, affecting its protein expression, and further regulates β‐catenin, PI3K, AKT1, and mTOR pathways to promote the development of ovarian cancer and epithelial ovarian cancer tumorigenesis and progression. Besides, SNORA70E also regulates the alternative splice of *PARPBP* to promote the development of ovarian cancer.

## AUTHOR CONTRIBUTIONS

**Shuo Chen:** Conceptualization (equal); data curation (equal); formal analysis (equal); funding acquisition (equal); methodology (equal); writing – original draft (equal); writing – review and editing (equal). **Qian‐hui Li:** Investigation (equal); methodology (equal). **Xi Chen:** Investigation (equal); methodology (equal). **Hai‐juan Bao:** Investigation (equal). **Wu Wu:** Investigation (equal). **Fan Shen:** Investigation (equal). **Bing‐feng Lu:** Formal analysis (equal); investigation (equal). **Ru‐qi Jiang:** Investigation (equal). **Zhi‐Hong Zong:** Funding acquisition (equal); methodology (equal). **Yang Zhao:** Conceptualization (lead); funding acquisition (equal); writing – review and editing (lead).

## CONFLICT OF INTEREST

The authors declare no conflict of interests.

## Supporting information


Table S1
Table S2.

Figure S1

Figure S2

Figure S3

## Data Availability

The resources, tools and codes used in our analyses were described in the methods section. The datasets generated during and/or analysed during the current study are available from the corresponding author on reasonable request. The public datasets of TCGA analysed during the current study is: https://portal.gdc.cancer.gov/projects/TCGA‐OV.
