# Supplementary material for: SNORA70E promotes the occurrence and development of ovarian cancer through pseudouridylation modification of RAP1B and alternative splicing of PARPBP
Source: J Cell Mol Med. 2022 Sep 3;26(20):5150–64. doi: 10.1111/jcmm.17540 (PMC9575132; doi:10.1111/jcmm.17540)
Supplement: Supplementary file 1 — Table S1 Table S2. [file JCMM-26-5150-s003.docx]

**Supplementary Table 1:** SNORA70E expression in ovarian tissues

| **Groups** | **N** | **SNORA70E expression / U6** | ***P* value** |
| --- | --- | --- | --- |
|  |  |  |  |
| Normal Ovary | 14 | 0.00295563 ± 0.00176448 | ***1.78E-07*** |
| Benign Tumors | 7 | 0.00304187 ± 0.00247689 | ***1.11E-06*** |
| Borderline Tumors | 12 | 0.00833571 ± 0.00574853 | ***0.009*** |
| Ovarian carcinoma | 70 | 0.01474697 ± 0.01722287 |  |

Bold and Italics means P < 0.05.

**Supplementary Table 2:** Correlation of SNORA70E expression with different clinicopathological features of ovarian carcinoma

| **Clinicopathological features** | **N** | **SNORA70E expression / U6** | ***P* value** |
| --- | --- | --- | --- |
|  |  |  |  |
| **The pathology types** |  |  | ***0.007*** |
| Serous carcinoma | 40 | 0.01872954 ± 0.02053607 |  |
| The other pathology types | 30 | 0.00943687 ± 0.00935773 |  |
| **Age** |  |  | 0.064 |
| ≤ 52 | 32 | 0.01149832 ± 0.00917897 |  |
| > 52 | 38 | 0.01748267 ± 0.02158159 |  |
| **FIGO stages** |  |  | ***0.045*** |
| I | 23 | 0.01052819 ± 0.01120060 |  |
| II-IV | 47 | 0.01681148 ± 0.01927920 |  |
| **Pathology classification** |  |  | ***0.021*** |
| Well | 22 | 0.01072806 ± 0.01138562 |  |
| Mod + Poor | 48 | 0.01658897 ± 0.01914491 |  |
| **CA125** |  |  | 0.232 |
| Negative | 15 | 0.01261991 ± 0.01031466 |  |
| Positive | 55 | 0.01532708 ± 0.01870403 |  |
| Bold and Italics means P < 0.05. | | | |
