# Supplementary figures and images for: SNORA70E promotes the occurrence and development of ovarian cancer through pseudouridylation modification of RAP1B and alternative splicing of PARPBP
Source: J Cell Mol Med. 2022 Sep 3;26(20):5150–64. doi: 10.1111/jcmm.17540 (PMC9575132; doi:10.1111/jcmm.17540)

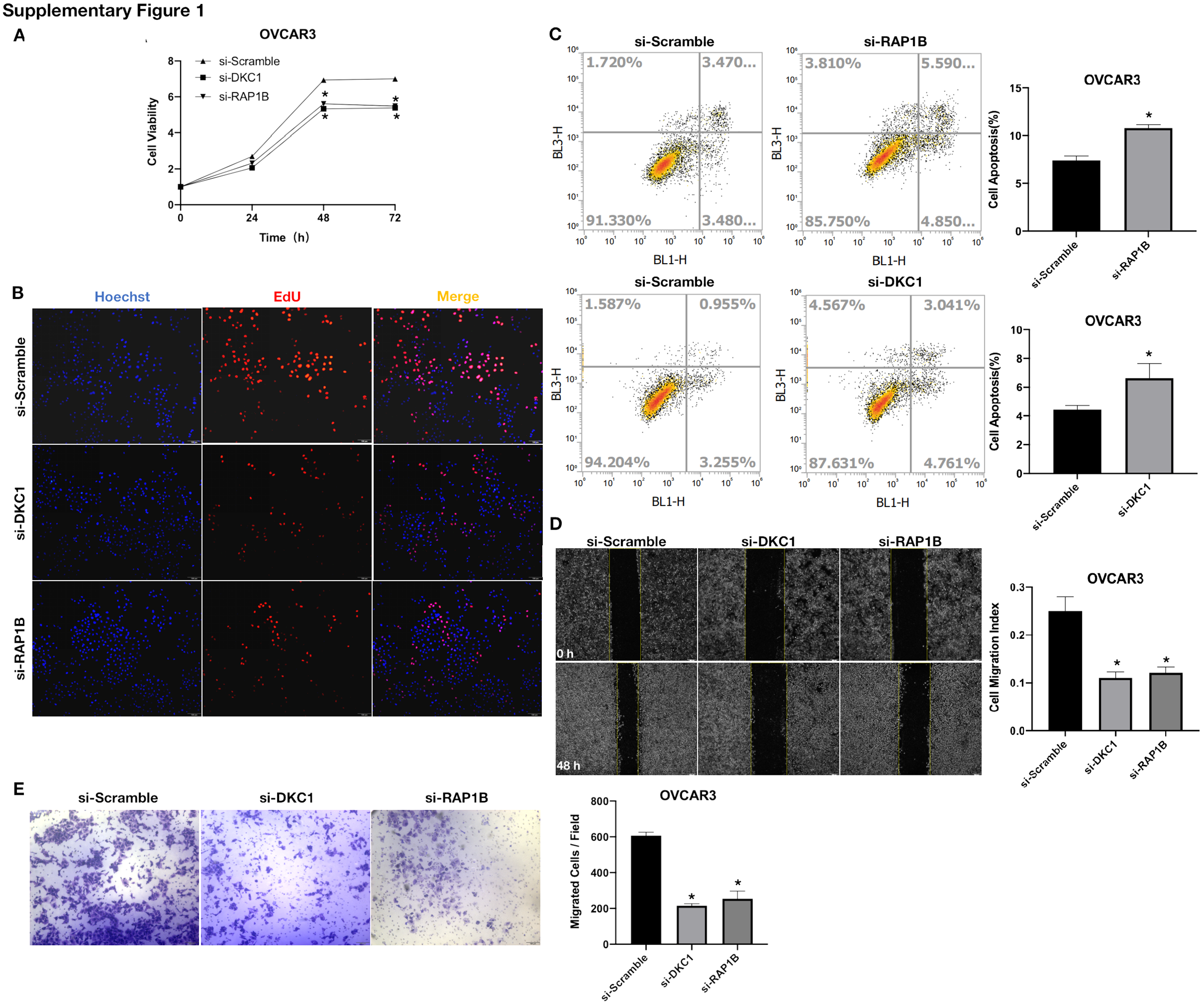

Supplement: Supplementary file 2 — Figure S1 [file JCMM-26-5150-s002.tif]

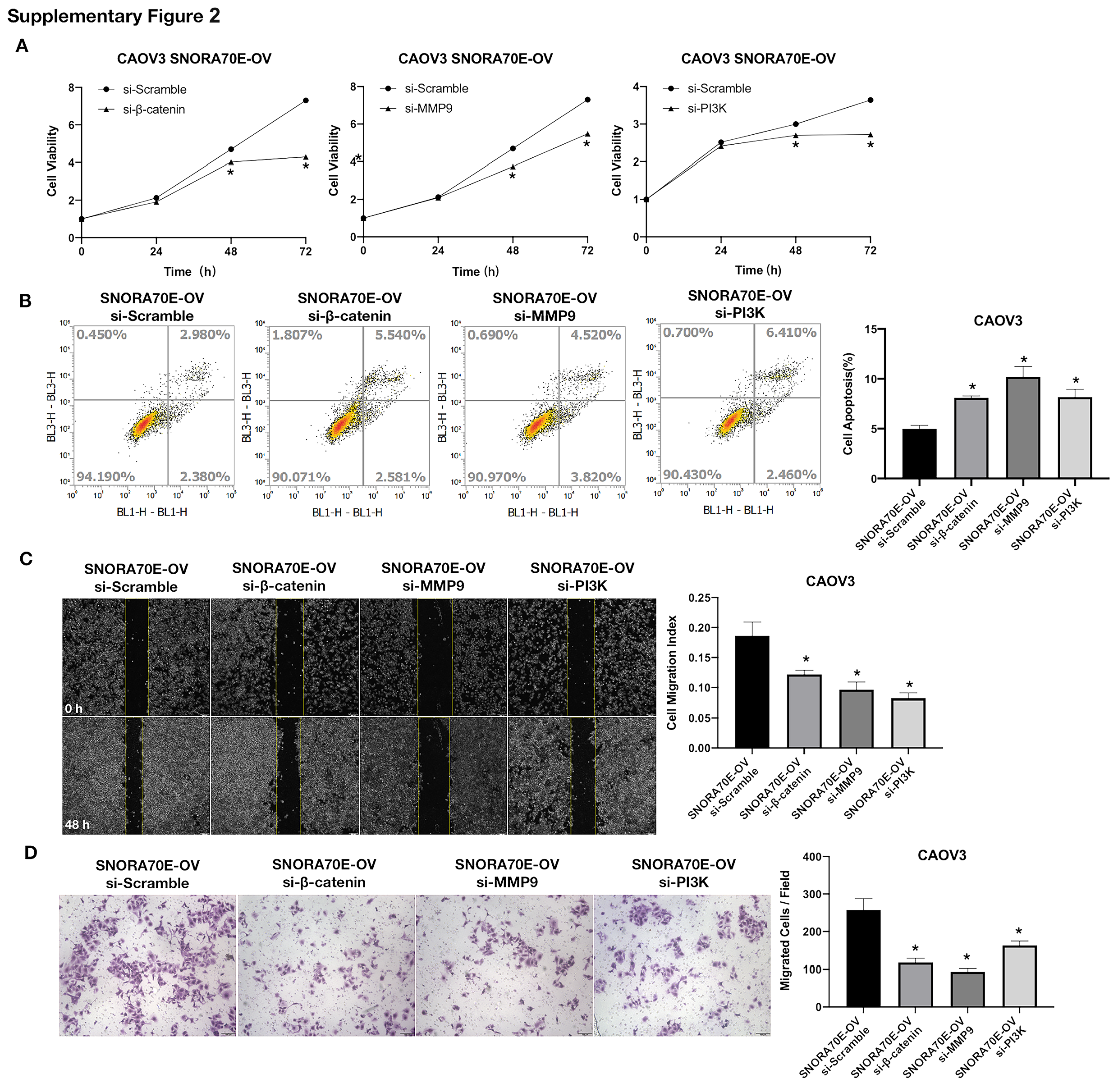

Supplement: Supplementary file 3 — Figure S2 [file JCMM-26-5150-s001.tif]

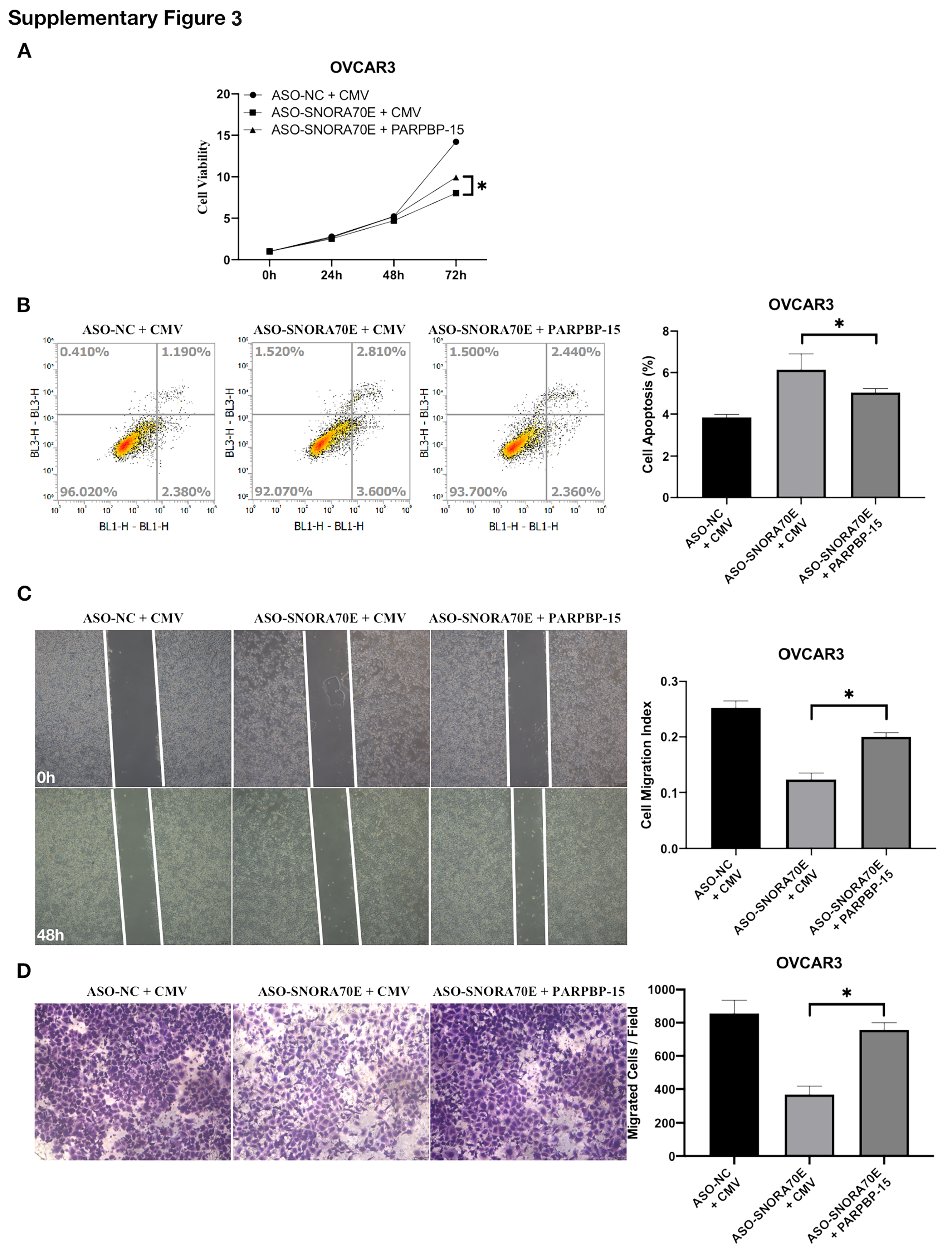

Supplement: Supplementary file 4 — Figure S3 [file JCMM-26-5150-s004.tif]
